# Venetoclax resistance: mechanistic insights and future strategies

**DOI:** 10.20517/cdr.2021.125

**Published:** 2022-05-06

**Authors:** Faustine Ong, Kunhwa Kim, Marina Y. Konopleva

**Affiliations:** Department of Leukemia, The University of Texas MD Anderson Cancer Center, Houston, TX 77030, USA.

**Keywords:** Venetoclax, acute myeloid leukemia, hypomethylating agents, Azacitidine, Decitabine, resistance, BCL2 protein, human

## Abstract

Acute myeloid leukemia (AML) is historically associated with poor prognosis, especially in older AML patients unfit for intensive chemotherapy. The development of Venetoclax, a potent oral BH3 (BCL-2 homology domain 3) mimetic, has transformed the AML treatment. However, the short duration of response and development of resistance remain major concerns. Understanding mechanisms of resistance is pivotal to devising new strategies and designing rational drug combination regimens. In this review, we will provide a comprehensive summary of the known mechanisms of resistance to Venetoclax and discuss Venetoclax-based combination therapies. Key contributing factors to Venetoclax resistance include dependencies on alternative anti-apoptotic BCL-2 family proteins and selection of the activating kinase mutations. Mutational landscape governing response to Venetoclax and strategic approaches developed considering current knowledge of mechanisms of resistance will be addressed.

## INTRODUCTION

Acute myeloid leukemia (AML) is the most common type of acute leukemia in adults^[1]^. The disease often affects older adults with a median age of 68 years at diagnosis^[1]^. Conventional treatments using intensive chemotherapy are less beneficial for older patients with poor tolerance and modest outcome^[2,3]^. Introduction of Venetoclax (ABT-199), a selective inhibitor of BCL-2, has advanced the treatment options for AML patients, especially older patients^[4]^. Venetoclax in combination with hypomethylating agents (HMA) or low-dose cytarabine (LDAC) was approved by US FDA for newly diagnosed AML patients who are either unable to receive intensive chemotherapy or older than 75 years old. Nowadays, this has become the standard therapy for such a population^[5]^. However, the prevalent use of Venetoclax comes with a new challenge of resistance, particularly in the relapsed/refractory setting^[6,7]^. Here, we review the mechanisms of Venetoclax resistance in AML and discuss strategies to overcome resistance.

## MECHANISM OF ACTION OF VENETOCLAX

### BCL-2 family proteins and mechanism of action of Venetoclax 

Venetoclax (ABT-199) is an oral, selective antagonist of the B-cell lymphoma 2 (BCL-2), a key protein modulating intrinsic (mitochondrial) apoptosis^[8]^. Apoptosis is regulated and balanced by protein-protein interactions among BCL-2 family members^[9]^. Different members of the BCL-2 family share BCL-2 homology motifs (BH1 to BH4)^[9]^. Anti-apoptotic proteins (BCL-2, BCL2A2, MCL-1, and BCL2L1 (BCL-xL), BCL-w, BFL-1/A1) sequester pro-apoptotic proteins by binding to its BH3 motifs^[9-13]^. Pro-apoptotic proteins consist of BH3-only proteins and effector proteins, BAK and BAX, which have BH1-4 motifs. BH3-only proteins act as sensitizers (BAD, BIK, HRK, NOXA) or activators (BIM, BID, PUMA) of apoptosis^[10,11,13]^. BH3-only sensitizer proteins are unable to activate downstream effector proteins (BAX, BAK) directly. However, they are able to “sensitize” cells toward apoptosis by binding to BCL-2 anti-apoptotic protein, releasing bound BAX or BAK or BH3-only activator protein^[14]^. Upon activation by bound BH3-only activator proteins, effector protein oligomerizes, leading to increase mitochondrial outer membrane permeabilization (MOMP) and initiation of cytochrome *c* mediated intrinsic apoptosis^[9-13]^. 

Dysregulation and imbalance of BCL-2 family members controlling apoptosis are commonly found in multiple hematologic malignancies^[12]^. Likewise, the BCL-2 family has an essential role in mediating AML survival and chemoresistance. Previous studies have demonstrated that an increase in the level of anti-apoptotic proteins, including BCL-2, is associated with chemotherapy resistance^[15-17]^. BCL-2 also supports the survival of leukemic stem cells in AML, and its inhibition induces the death of quiescent leukemic stem cells^[18]^. Hence, BCL-2 is an important therapeutic target. Venetoclax, a BH3 mimetic, binds to a BH3-binding groove of BCL-2 protein with high selectivity^[8]^. This relieves inhibition of BCL-2 toward BAX and BAK, resulting in cell death^[9]^. Preclinical studies demonstrated high anti-tumor activity of Venetoclax in AML, which facilitated further clinical studies^[16,19]^. In an initial phase 2 study, Venetoclax monotherapy produced modest responses in heavily treated relapsed or refractory (R/R) AML patients with an overall response rate (ORR) of 19%^[4]^. As AML may not depend on BCL-2 for its survival or the dependency may evolve during tumor progression and after therapy^[20]^, a method to assess BCL-2 family dependency is critically needed to predict sensitivity to Venetoclax^[4,13,21,22]^. BH3 profiling is performed by exposing mitochondria to a specific BH3 peptide, followed by measurement of cytochrome *c* release and MOMP^[23]^. Addiction to a specific BCL-2 anti-apoptotic protein is inferred from cellular apoptotic sensitivity when exposed to different BH3 peptides^[23]^.

### Genomic biomarkers associated with Venetoclax sensitivity 

Early phase 2 study on Venetoclax monotherapy suggested spliceosomal mutation in SRSF2/ZRS2 and IDH1/2 as predictors for Venetoclax sensitivity^[4]^. Ten out of 11 patients with baseline SRSF2 or ZRSR2 mutation had a measurable reduction in bone marrow (BM) blast after treatment with Venetoclax^[24]^. *SRSF2* genes were found to induce alternative splicing of apoptosis regulating genes and modulate the expression of BCL-2 family proteins, which may increase sensitivity to Venetoclax^[24,25]^.

In preclinical models, IDH mutation in AML conferred high BCL-2 dependence and sensitivity to BCL-2 inhibition. The mechanism involved oncometabolite (R)-2 hydroxyglutarate (2-HG), which inhibits mitochondrial cytochrome *c* oxidase, causing a reduction of mitochondrial threshold for apoptosis induction^[26]^. A recent study by Stuani *et al.*^[27]^ described additional mechanisms behind IDH mutant sensitivity to BCL-2 inhibitor through inhibition of mitochondrial respiration. Mutant IDH cells and patient-derived xenografts (PDX) were found to have a larger capacity for mitochondrial oxidative phosphorylation (OXPHOS), as evident by increased electron transport chain (ETC) complex I activity, NADH production by tricarboxylic acid cycle enzymes, mitochondrial ATP content and oxygen consumption rate^[27]^. Gene set enrichment analysis on IDH1 mutant cells also showed enhanced fatty acid oxidation (FAO) gene signature, especially CPT1a, an acyl-carnitine transporter during FAO, and its transcriptional regulator, CEBPα^[27]^. While 2-HG was shown to drive CPT1a and CEBPα-dependent FAO and OXPHOS, abrogation of 2-HG production by IDH inhibitor did not impact FAO rate or OXPHOS in the treated AML cell lines, suggesting maintenance of OXPHOS phenotype independent of 2-HG^[27]^. Inhibition of OXPHOS and IDH1 mutation subsequently showed synergistic cytotoxic activity and improved cell differentiation *in vitro *and *in vivo*^[27]^.

Venetoclax, a BCL-2 inhibitor, was reported to target leukemia stem cells (LSCs) metabolism through reduction of ETC complex II activity and OXPHOS^[28]^. Consistent with preclinical data, IDH mutant cells showed heightened sensitivity upon OXPHOS inhibition by Venetoclax therapy^[27]^. In the phase 2 trial of a single agent Venetoclax, IDH1/2 mutated R/R AML subset reached a higher objective response rate of 33% as compared to 10% ORR in the IDH-wildtype AML subset^[4]^. Mirroring synergy seen in preclinical study^[27]^, a preliminary analysis from an ongoing phase 1/2 clinical trial demonstrated encouraging composite complete remission of 75% with median overall survival (OS) of 9.7 months in the R/R cohort upon treatment with a combination of Ivosidenib (an IDH1 inhibitor), Venetoclax and Azacitidine “triplet”^[29]^. Future updates are anticipated to evaluate clinical characteristics and molecular predictors of response. In parallel, a phase 1b/2 study of Enasidenib (an IDH2 inhibitor) and Venetoclax combination is underway (NCT04092179) after demonstrated efficacy in preclinical studies^[30,31]^.

Utilizing highly sensitive quantitative reverse transcription polymerase chain reaction (RT-qPCR), a clinical study by DiNardo *et al.*^[32]^ found a strikingly high rate of mutation clearance in NPM1 mutated patients upon treatment with a combination of Venetoclax and HMA or LDAC (4/4 cases). As expected, a high molecular remission rate translated into an excellent survival with relapse-free survival not reached after a median follow-up of 20 months in patients who initially had persistent mutation despite intensive chemotherapy^[33]^. Unlike IDH mutated AML, the mechanistic basis of Venetoclax sensitivity in NPM1 mutated AML is not well understood^[32]^. However, clinical findings indicate NPM1 (without FLT3 co-mutation) as an important predictor of Venetoclax sensitivity.

ASXL1 mutation was recently found to drive response to Venetoclax *in vitro*. ASXL1 acts as an epigenetic regulator via PRC2-mediated chromatin modification to keep various genes in a repressed state^[34]^. Thus, ASXL1 mutation leads to aberrant activation of its target genes, including BCL2^[34]^. In preclinical study of Rahmani *et al*.^[34]^, ATAC-sequencing analysis suggested higher chromatin accessibility on the BCL-2 locus of ASXL1 mutant KBM5 cells due to failure of PRC2 complex recruitment, resulting in BCL-2 overexpression^[34]^. BH3 profiling further showed higher BCL2 anti-apoptotic protein dependency in ASXL1 mutant cells, which explained ASXL1 sensitivity to Venetoclax *in vitro*^[34]^. Rahmani *et al.*^[34]^ also demonstrated enhanced global cytosine methylation, which led to sensitivity to Azacitidine, a DNMT inhibitor. Thus, treatment of ASXL1 mutant cells with single agent Venetoclax or Azacitidine resulted in increased cell differentiation, decreased cell growth and viability^[34]^. Analysis of data from clinical trials is needed to prove this finding in the clinical setting.

## VENETOCLAX RESISTANCE: PRECLINICAL STUDIES

### Cellular mechanism of resistance to Venetoclax: other BCL-2 family proteins

**Figure 1:**
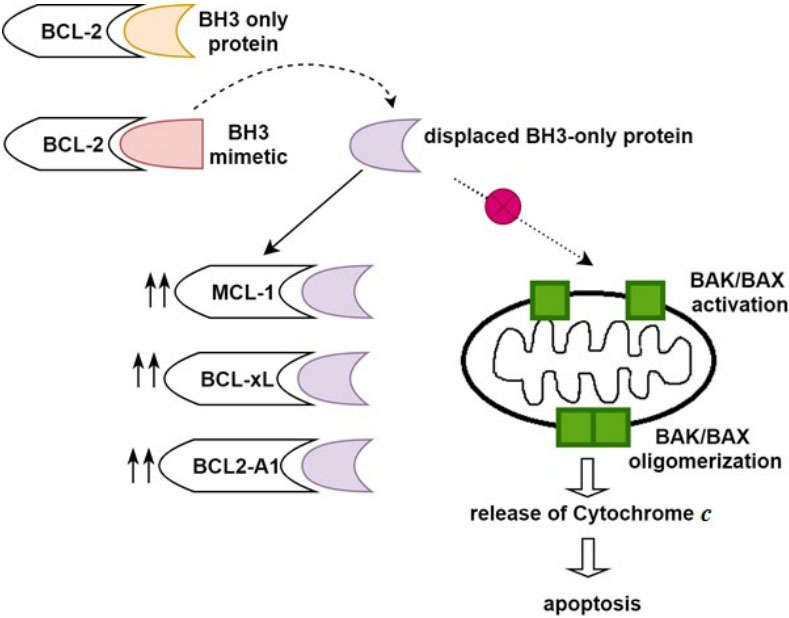
Binding of BH3 mimetic, Venetoclax to BCL-2 anti-apoptotic protein releases bound BH3-only protein, subsequently allowing interaction between BH3-only protein and BAK/BAX. Upregulation of MCL-1, BCL-xL, and BCL2-A1 confers Venetoclax resistance by sequestration of BH3 only proteins, preventing them from interacting with BAK/BAX and avoidance of apoptosis^[35-40]^.

Dependencies on other anti-apoptotic BCL-2 family members, including BCL2-A1, MCL-1, and BCL-xL, have been demonstrated as the key contributors to primary or adaptive Venetoclax resistance^[4,7,13,35-37]^. Venetoclax resistance is associated with sequestration of BIM freed from Venetoclax binding to BCL-2 by other Bcl-2 family members, which consequently prevents apoptosis [Figure 1]^[38,39]^. Preclinical work demonstrated lower expression of MCL-1 and BCL-xL in Venetoclax sensitive AML cell lines and lower BCL-2 protein levels in resistant cells^[13]^. When resistance was induced in AML cell lines through exposure to Venetoclax over several months, a shift was seen toward upregulation of MCL-1 and or BCL-xL with less dependency on BCL-2^[35,40]^. To investigate this potential mechanism, BCL-xL and MCL-1 were inactivated with BCL-xL inhibitor (WEHI-539) and short hairpin RNA (shRNA), respectively, which resulted in the restoration of Venetoclax sensitivity in resistant AML cell lines^[35]^. Utilizing BH3 profiling, a phase 2 study of Venetoclax monotherapy for R/R AML showed shorter durability of Venetoclax responses in patients with MCL-1 or BCL-xL dependence, suggesting that these mechanisms are operational in the clinical setting^[4]^.

The knowledge around common co-dependencies in leukemia and cancer led to the exploration of selective BCL-xL and MCL-1 inhibitors. Navitoclax (ABT263), a predecessor of Venetoclax, possessed a high affinity for BCL-2, BCL-xL and BCL-w^[41]^. However, its clinical use has been limited by on-target toxicity of thrombocytopenia due to reliance on BCL-xl for platelet survival^[42]^. Recently there is a growing interest in the exploration of MCL-1 inhibitors, which have shown striking synergy with BCL-2 inhibitors in resistant AML cell lines and xenograft models^[40,43,44]^. Multiple MCL-1 inhibitors are under development, and several are being evaluated in clinical trials^[45-47]^.

In recent studies, transcriptomic analysis of AML patients’ samples showed differential expression of BCL2A1 in resistant cells^[36,37]^. Upregulation of BCL2A1 expression correlated with higher Venetoclax’s area under the curve (AUC) and less apoptosis after treatment with Venetoclax with or without Azacitidine and Cytarabine. Knockdown of BCL2A1 restored apoptosis and reduced cell growth in the resistant AML cells without substantial effect on the CD34+ hematopoietic stem and progenitor cell (HSPC)^[37]^. Therefore, there is a potential for synergy between Venetoclax and BCL2A1 inhibitor in selective AML subsets, similar to Venetoclax and MCL-1 inhibitor^[37,44]^.

### Mutations in activating kinases and Venetoclax resistance

Activation of intracellular signaling pathways by KRAS/PTPN1 or FMS-like tyrosine kinase 3 (FLT3) mutant proteins is postulated to induce Venetoclax resistance. Genomic biomarkers were analyzed before and after treatment with Venetoclax monotherapy^[24]^. Three out of 14 patients with pre-treatment FLT3-internal tandem duplication (ITD) and 4 out of 14 patients with PTPN11 mutation failed to achieve bone marrow blast reduction, suggesting intrinsic resistance to Venetoclax. In addition, a subset of patients at the time of relapse were found to harbor FLT3-ITD and/or PTPN11 mutations not identified prior to therapy, strongly indicating the emergence or selection of these mutations as secondary or acquired resistance^[24]^.

**Figure 2:**
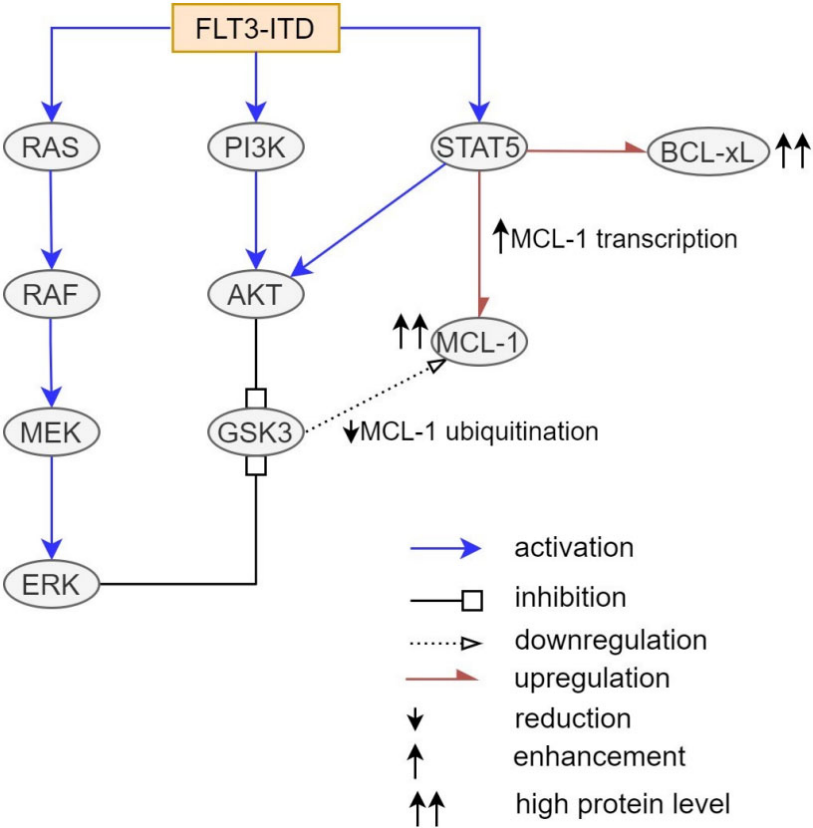
FLT3-ITD mutation causes an increased level of BCL-xL and MCL-1 via activation of downstream PI3K-AKT, RAS-MAPK, and STAT5 pathways. AKT and ERK promoted inhibitory phosphorylation of GSK3, leading to a reduction of MCL1 ubiquitination and degradation. In addition to upregulation of MCL-1 and BCL-xL, STAT5 also increases MCL-1 indirectly through AKT activation. In summary, FLT3-ITD mutation confers Venetoclax resistance by upregulation of BCL-xL and MCL-1^[48-53]^.

FLT3-ITD mutation occurs in about 25% of adult AML cases and confers an adverse prognosis^[27]^. FLT3-ITD mutation promotes survival via activation of PI3K-protein kinase B (Akt), RAS-MAPK, and STAT5 pathway^[14,38]^. While the precise downstream molecular pathways are yet to be defined, FLT3-ITD mutation is known to induce higher expression of BCL-xL and MCL-1, which may contribute to Venetoclax resistance [Figure 2]^[48-50]^. Several studies demonstrated the involvement of the molecular pathway downstream of FLT3 in modulating BCL-xL and MCL-1 expression. STAT5, which was activated by FLT3-ITD, but not by their wild-type counterpart^[50]^, was found to regulate transcription of the *BCL-xL* gene^[51]^. Akt, a downstream substance of PI3K pathway, was shown to influence MCL-1 stabilization by inactivation of glycogen synthase kinase 3 (GSK3), leading to sequestration of BIM and prevention of MOMP^[52]^. Yoshimoto *et al.*^[50]^ further reported the role of STAT5 and Akt in MCL-1 upregulation. Suppression of STAT5 by small interfering RNA (siRNA) reduced the level of MCL-1 protein and mRNA in FLT3-ITD^+^ MV4-11 cell lines. In addition to direct stimulation of MCL-1 transcription, STAT5 also enhances phosphorylation of Akt, which indirectly increases MCL-1 expression in FLT3-ITD cells^[50]^. 

Several preclinical studies evaluated the efficacy of combining FLT3 inhibitor and Venetoclax in FLT3-ITD^+^ cell lines, patient samples, and xenograft models. Ma *et al.*^[38]^ assessed the efficacy of Midostaurin (1^st ^generation type 1 FLT3 inhibitor)^[3]^ and Gilteritinib (2nd generation type 1 FLT3 inhibitor)^[3]^ in combination with Venetoclax. Midostaurin or Gilteritinib in combination with Venetoclax synergistically induced apoptosis in FLT3-ITD+ cell lines and patient samples^[38]^. The combination of Gilteritinib and Venetoclax also resulted in higher survival of the MV4-11 xenograft model compared to either drug alone, with four out of five treated mice remaining disease-free on day 190^[38]^. A similar finding was reported by Zhu *et al.*^[14]^ The combination of Gilteritinib with Venetoclax outperformed their respective monotherapy in halting the proliferation of FLT3-ITD^+^ cells and reducing tumor burden in the FLT3-ITD^+^ patient-derived xenograft (PDX) and resistant MOLM-14 model^[14]^. Another set of experiments with a combination of 2nd generation type 2 FLT-3 inhibitor (Quizartinib)^[3]^ and Venetoclax *in vitro *and* in vivo*, resonated with prior studies^[49]^. Co-treatment with Quizartinib and Venetoclax in the xenograft model produced a longer response with a delay in tumor resurgence for up to three months post-treatment^[24]^.

In these studies, mechanisms driving the synergy between FLT3 inhibitor and Venetoclax were interrogated. Ma *et al.*^[38] ^and Zhu *et al.*^[14]^ demonstrated that treatment with FLT3 inhibitor (Midostaurin or Gilteritinib) alone or in combination with Venetoclax reduced the expression of MCL-1 *in vitro*^[14,38]^. Utilizing western blot, reduced phosphorylation of ERK, AKT, STAT5 was seen after 24 hours of treatment with Gilteritinib or Quizartinib. This finding suggests FLT3 inhibition modulates MCL-1 by simultaneous suppression of multiple kinase pathways including RAS-MAPK, PI3K-AKT, and STAT5^[14,38]^. Co-immunoprecipitation assay in FLT-ITD^+^ cell lines further revealed decreased binding of BIM to MCL-1 and increased binding of BIM to BCL-2 after Gilteritinib treatment, while the opposite was seen with Venetoclax treatment^[14,38]^. Interestingly, co-treatment with Gilteritinib and Venetoclax also increased the binding of BIM to BAX without increasing BIM binding to other BCL-2 anti-apoptotic proteins, specifically BCL-xL^[14]^. Thus, combination therapy with FLT3 inhibitor and Venetoclax *in vitro *reduced BIM binding to both BCL-2 and MCL-1, liberating BIM to interact with BAX and induce apoptosis^[14]^.

**Figure 3:**
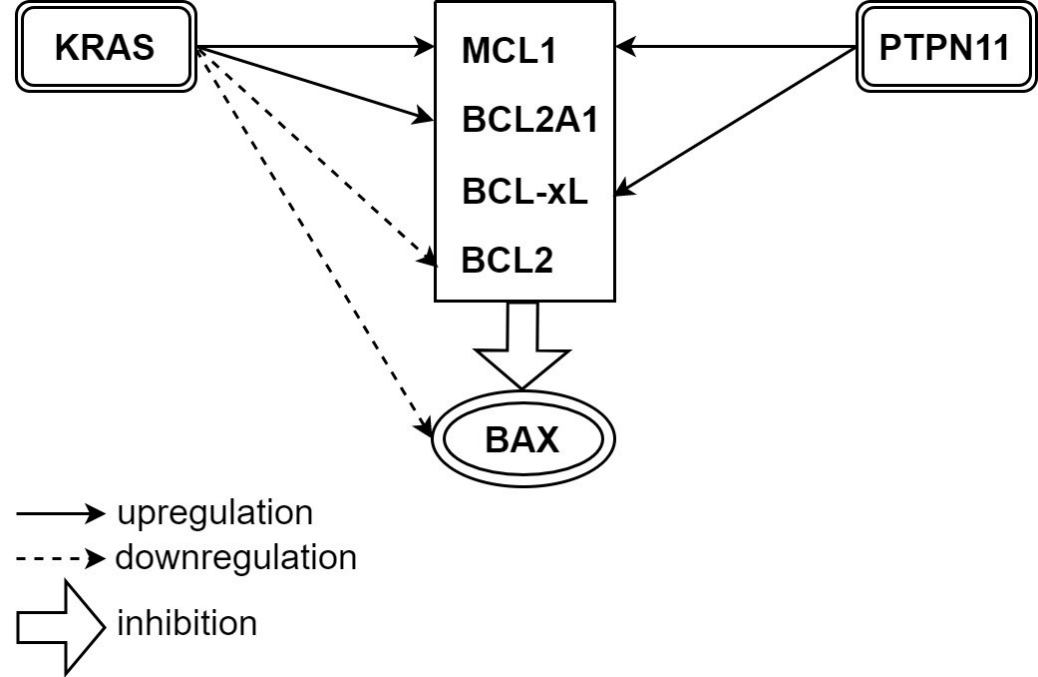
Both KRAS and PTPN11 mutations confer Venetoclax resistance. KRAS mutation causes upregulation of MCL-1 and BCL2A1, while PTPN11 mutation causes upregulation of MCL-1 and BCL-xL. KRAS mutation also downregulates BCL-2 and BAX^[37]^.

In addition to FLT3, mutations in other activating kinases can confer resistance to Venetoclax (so-called “signaling” mutations). Genomic data from primary patient samples in the BEAT AML database was analyzed along with Venetoclax AUC established *in vitro*^[37]^. Samples with KRAS or PTPN11 mutations were found to have higher Venetoclax AUC. Venetoclax resistance was reproduced when AML cell lines were transduced to overexpress G12D KRAS and A72D PTPN11^[37]^. Analysis with RT-PCR and immunoblot showed decreased BCL-2 and BAX with increased MCL-1 and BCL2A1 levels in G12D KRAS cells, as well as increased MCL-1 and BCL-xL levels in A72D PTPN11 cells [Figure 3]^[37]^. G12D KRAS cells showed a reduction in cellular viability after treatment with MCL-1 inhibitors (AZD5991)^[37]^. However, neither BCL-2 blockade nor simultaneous blockade of BCL-2, BCL-w, and BCL-xL by ABT263 and ABT737 produced a similar response^[37]^. This finding implied KRAS mutation dependency on MCL-1 to drive Venetoclax resistance. In A72D PTPN11, AZD5991 was also capable of suppressing PTPN mutant cells, while partial response only was seen with BCL-2/Bcl-XL dual inhibitors ABT-263 and ABT 737. This finding suggests partial dependence of PTPN-induced Venetoclax resistance on MCL-1 and BCL-xL^[37]^. As expected, the combination of AZD5991 and Venetoclax showed synergy and fully rescued mutant cell lines from KRAS- and PTPN11-mediated resistance. Hence, combinatorial therapy with Venetoclax and MCL-1 inhibitor is expected to demonstrate high efficacy in AML patients harboring these mutations^[37]^.

### Roles of TP53, BAX, and mitochondria in Venetoclax resistance

In a recent report, BAX variants were found by deep sequencing performed on samples derived from AML patients who relapsed after initially achieving remission with Venetoclax-based regimens, signifying acquired BAX mutation as adaptive Venetoclax resistance^[54]^. Reduced survival was also seen when BAX deficient OCI-AML3 cells were transplanted into the AML xenograft model^[54]^. BAX deficient cells and xenograft model were resistant to cell death induced by Venetoclax, MCL-1 inhibitor (S63845), or a combination of both^[54]^. This is contrary to a prior study which showed sensitivity of BAX knockout (KO) cells to a different MCL-1 inhibitor (AZD-5991) with similar resistance to Venetoclax and BCL-2/BCL-xL inhibitor (AZD-4320)^[55]^. Hence, particular attention to BAX mutant subsets is warranted in future studies to evaluate its impact on response to BH3 mimetics as single agents or combinations.

Genome-scale CRISPR/Cas9 screening identified BAX along with TP53 and PMA1P1 (NOXA) as genes whose inactivation confers Venetoclax resistance^[40,55,56]^. Gene enrichment and protein-protein interaction analysis identified these genes as an essential part of the mitochondrial apoptosis pathway^[55,56]^. TP53 is activated by cellular stress and functions as a transcription factor for genes controlling various cellular processes, including apoptosis and cell cycle arrest^[55]^. Several BH3-only proteins, including *BAK, BAX, PUMA, *and* NOXA*, are also TP53 target genes^[55,57,58]^. As expected, a lower level of BAK1, PUMA, and NOXA was observed in TP53 KO cells^[55]^. Interestingly, transcriptional changes were observed outside TP53 target genes with an increased ratio of BCL-xL/BCL-2, which may further confer Venetoclax resistance in TP53 KO cells^[55]^. 

Several preclinical studies demonstrated that TP53 mutated cells and xenograft models are resistant to single-agent therapy with Venetoclax^[55,59]^ or MCL-1 inhibitor^[55,59,60]^. Contesting these findings, Thijssen *et al.*^[60] ^reported observation that the lack of TP53 function did not preclude cell killing by sub-lethal BCL-2 or MCL-1 inhibitor monotherapy. However, this response was not durable as surviving TP53 deficient cells outgrew TP53 wild-type cells over a longer period of exposure, indicating a competitive survival advantage^[60]^. Delayed activation of BAX/BAK and subsequent apoptosis upon BH3 mimetics treatment was thought to cause reduced BH3 mimetics efficacy by lifting the early apoptosis threshold in the absence of TP53. Notably, simultaneous inhibition of BCL-2 and MCL-1 was able to overcome the apoptotic delay with improved durability of response^[60]^, resonating with synergistic action described in past studies^[55,59]^.

The impact of TP53 and BAX on mitochondrial function and morphology was evaluated. TP53 and BAX KO cells demonstrated less mitophagy when exposed to stress by a mitochondrial uncoupler and increased cellular respiration, reflected by higher production of cellular reactive oxygen species (ROS)^[55]^. Furthermore, TP53 and BAX KO cells also showed aberrant metabolic profiles with increased nucleotide synthesis and parallel decreases in glucose, pyruvate, amino acids, and urea cycle intermediates levels, suggesting priority shifting on carbon usage to support cancer cell proliferation^[55]^. These findings highlight the crucial role of mitochondria in mediating sensitivity or resistance to Venetoclax.

A deeper dive into mitochondrial biology found that aberrant mitochondrial architecture and bioenergetics were implicated in the apoptotic response to Venetoclax^[56]^. CRISPR/Cas9 screening further identified a negative association between Venetoclax resistance and mitochondrial chaperonin CLPB, which regulates mitochondrial cristae and metabolism^[56]^. Chen *et al.*^[56]^ reported overexpression of CLPB in AML which led to tighter mitochondrial cristae lumen. On the other hand, loss of CLPB resulted in wider crista and stimulation of mitochondrial stress response, which in turn triggered cell cycle arrest and lowered the mitochondrial threshold for apoptosis^[56]^. A combination of CLPB deletion and Venetoclax was found to rescue Venetoclax resistant AML cells even in the presence of TP53 mutation^[56]^. Another genomic CRISPR-Cas9 knockout screen demonstrated a negative selection of *DAP3*, *MRPL54*, *MRPL17*, *RBFA* genes, which are part of the mitochondrial translation machinery^[61]^. When mitochondrial protein synthesis was blocked by tedizolid or doxycycline, reduction of AML cell viability was only observed upon co-treatment with Venetoclax, but not with Venetoclax or tedizolid or doxycycline alone^[61]^. A subsequent investigation by Sharon *et al.*^[61] ^demonstrated that the combination of Venetoclax and tedizolid led to augmentation of the integrated stress response with associated reduction of OXPHOS, and decreased glycolytic capacity, resulting in ATP consumption and cell death.

### Monocytic AML in Venetoclax resistance

Several preclinical studies suggested that AML may respond differently to Venetoclax-based therapies, depending on the blast differentiation stage. In the studies discussed below, AML was classified based on cell morphology according to the French, American, and British (FAB) classification system^[62]^. Guided by flow cytometry, ex vivo drug sensitivity testing on patient samples with primary AML showed a progressive increase in Venetoclax resistance through the cell maturation phase from the most primitive AML (M0) to monocytic AML (M5)^[63,64]^. Aligned with this observation, analysis of the BEAT AML dataset also noticed higher Venetoclax AUC in the leukemic blasts with high expression of CD14 and CLEA7A (gene encoding CD369) that are usually present in M4/M5 AML, suggesting Venetoclax resistance in AML with myelomonocytic or monocytic differentiation^[37]^.

Further analysis also indicated different expression levels of alternative BCL-2 anti-apoptotic protein at the selected stages of cell maturation, which correlates with Venetoclax resistance. For instance, a gradual decline in BCL-2 expression with a concurrent increase in MCL-1 expression from M0 to M5 was observed, suggesting a lineage-associate switch to MCL-1 in monocytic AML^[63,64]^. In addition, the link between high expression of CD14 and CLEA7A, presence of KRAS mutation and increased BCL2A1 were seen in M4/5 AML^[37,63,64]^. Hence, Venetoclax resistance in AML with myelomonocytic differentiation may additionally be governed by BCL2A1 through upstream mutant KRAS^[37,63]^.

Consistent with these laboratory predictions, analysis of matched patient samples at diagnosis and relapse post-Venetoclax revealed the coexistence of primitive and monocytic features at diagnosis, suggesting developmental heterogenicity within the leukemic blast^[64]^. Monocytic clone subsequently expanded under the selective pressure of Venetoclax and Azacitidine, with the contemporaneous vanishing of primitive population at the time of relapse^[64]^. Interestingly, a portion of relapsed monocytic subclone also showed increased HOXA9 expression (similar to that observed in MLL-rearranged leukemia)^[65]^, which was not present in their parental clone^[64]^. However, both diagnosis and relapsed monocytic clone retained their MCL-1 dependency^[64]^. These findings suggest the potential role of menin inhibitor or MCL-1 inhibitor to overcome Venetoclax resistance in monocytic disease^[64,65]^.

## VENETOCLAX-BASED COMBINATION THERAPIES: LESSONS FROM CLINICAL STUDIES

### Venetoclax-based combination therapies

Given low response rates to Venetoclax as a single agent in R/R AML Phase II study, further studies focused on combination regimens. Several lines of clinical trials investigated the combination of Venetoclax with HMA or low-dose cytarabine in both frontline and R/R settings^[66-69]^. Monotherapy with HMA was commonly used in unfit or older patients with AML, but its benefit is limited by low response rates of ~30% and modest median survival at 8-10 months^[70-73]^. The synergy between Venetoclax and HMA therapy was reported in preclinical studies. Bogenberger *et al.*^[74,75]^ demonstrated increased sensitivity of HMA after inhibition of BCL-2 family proteins in AML samples. Tsao *et al.*^[76]^ suggested Azacitidine has activity against MCL-1, and it induces synergism with Venetoclax. Subsequent clinical trials validated the clinical efficacy of these regimens.

In a phase 1b study, Venetoclax was combined with either Decitabine or Azacitidine to treat 145 older, newly diagnosed patients unfit for induction chemotherapy. Sixty-seven percent of patients achieved CR/CRi, including 60% of CR/CRi rates in patients with poor cytogenetics risk. The median OS in the study was 17.5 months, and the median duration of response was 11.3 months, with a tolerable safety profile^[66]^. Following phase 2 study of 10-day decitabine and Venetoclax for intensive chemotherapy ineligible patients, including 70 newly diagnosed AML and 55 R/R AML, confirmed acceptable safety profile and high efficacy with CR/CRi rate of 74%. The study showed a median OS of 18.1 months in newly diagnosed de-novo AML and six months in R/R AML^[68]^. A randomized phase 3 clinical trial (VIALE-A) comparing Azacitidine monotherapy to combination therapy with Azacitidine and Venetoclax as a frontline treatment confirmed improved response rate and survival in Azacitidine/Venetoclax treated patients compared with Azacitidine alone. CR/CRi rate was 66% in the combination therapy arm compared to 28% in patients who received single Azacitidine^[77]^. OS was longer in the combination group at 14.7 months with a median event-free survival (EFS) of 9.8 months compared to 9.6 months of OS and seven months of EFS in the single-agent Azacitidine group.

Cytarabine, a commonly used cytotoxic chemotherapy agent in AML, was likewise shown to have synergism with Venetoclax through inhibition of MCL-1, increased BH3 activity, and upregulation of pro-apoptotic protein, Bim^[78]^. A phase 1b/2 study of low-dose Cytarabine (LDAC) combined with Venetoclax in newly diagnosed older AML patients demonstrated excellent safety data and a high response rate with CR/CRi of 54% and median OS of 10.1 months^[69]^. This is encouraging compared to results from prior clinical studies of low-intensity cytotoxic therapy, which showed response rates of less than 20% and median OS of less than six months. Thus, the combination of LDAC and Venetoclax showed significantly improved efficacy compared to conventional chemotherapy^[72,79]^. In a randomized phase 3 trial in 211 newly diagnosed AML patients ineligible for intensive treatment, the combination of Venetoclax and LDAC was compared to LDAC with placebo. The combination group had a higher CR/CRi rate of 48% compared to 13% of the control group, with an EFS of 4.7 months in the combination group compared to 2 months in the control group^[80]^. The combination group also had longer OS at 7.2 months compared to 4.1 months in the control group, with a 25% reduction in risk of death^[80]^.

In a phase 1b/2 study of Venetoclax with high-intensity chemotherapy FLAG-IDA (Fludarabine, Cytarabine, granulocyte colony-stimulating factor and Idarubicin), 68 younger AML patients with a median age of 46 years old were enrolled, with 29 de novo and 39 R/R patients^[81]^. The study demonstrated high clinical efficacy of the combined regimen with median survival not reached at the 12 months median follow-up, and a high minimal residual disease (MRD) negative CR rate of 96% in ND-AML patients and 69% in R/R AML patients. The majority of patients treated with this regimen proceeded towards allogeneic stem-cell transplant (69% of de novo AML and 46% of R/R AML patients)^[81]^.

In another phase 1b study, Venetoclax was given in a dose-escalated fashion up to 600 mg prior to Cytarabine and Idarubicin infusion (5 + 2) in fit older patients with AML age 60 years and above (CAVEAT study)^[82]^. The combination regimen was well tolerable and efficacious, with ORR of 97% and 43% in de novo AML and secondary AML, respectively. Median OS was 11.2 months, with longer median OS seen in de novo AML of 31.3 months and 29.5 months in patients who achieved CR^[82]^. This study proved the acceptable tolerability and high efficacy of Venetoclax when given in combination with standard intensive chemotherapy in fit older adults with AML^[82]^.

An ongoing phase 2 trial of the combination of Venetoclax with Cladribine plus LDAC alternating with hypomethylating agents in 48 newly diagnosed older AML patients demonstrated a high CR/CRi rate of 94% with MRD-negativity of 92%. Median OS was not reached over the 11-month median follow-up, and 24% of patients received an allogeneic stem-cell transplant^[83]^.

### Molecular landscape associated with response to Venetoclax combination therapies

Analysis of pre-treatment patients’ characteristics and paired AML samples pre-treatment and at the time of progression was performed to delineate the molecular patterns associated with treatment response to the combination of Venetoclax with HMA or with low dose Cytarabine (LDAC). In the frontline setting, AML patients with NPM1 or IDH1/2 mutations achieved high rates of CR/CRi and had sustained response for > 12 months^[32,66]^. Median duration of CR/CRi and median OS was not reached in IDH1/2 or NPM1 mutated patients^[66]^. Real-world experience supported the observations of IDH1/2 and NPM1 mutations as molecular predictors of response, even in the R/R setting^[84,85]^. 

It is noteworthy that deep molecular remissions were seen in patients with NPM1 mutation^[32,33]^. Highly sensitive RT-qPCR demonstrated MRD clearance in all recruited patients with NPM1 mutation upon treatment with Venetoclax and HMA or LDAC (4/4 cases)^[32]^. A subsequent study by Tiong *et al.*^[33]^ also reproduced this finding in NPM1 mutated patients with molecular persistence or relapse after treatment with standard intensive chemotherapy. In addition, there was no molecular or hematological relapse observed in NPM1 mutated patients who achieved MRD negative remission after a median follow-up of 11.8 months^[33]^. Notably, two of the older adult patients unfit for stem cell transplantation (SCT) in this study were alive at 6 and 12 months of follow-up^[33]^, validating Venetoclax-based therapy as a safe and well-tolerated therapy in the frail older adult population. This finding further highlights Venetoclax as a highly efficacious agent to treat NPM1 mutated AML even after intensive chemotherapy failure. A longer follow-up would be needed to evaluate the duration of MRD clearance with Venetoclax based therapy. If indeed durable, a Venetoclax-based regimen could be a well-tolerated and effective option in lieu of SCT in the unfit older population with NPM1 mutated AML. As deep remission was produced by Venetoclax-based therapy after failure of intensive chemotherapy treatment, further studies would be needed to assess Venetoclax-based therapy as a non-inferior or even a superior alternative to intensive chemotherapy in NPM1 mutated younger AML patients prior to transplantation.

Despite the general association with improved response to Venetoclax-based regimens, several points need to be taken into consideration with respect to NPM1 and IDH2 mutations. Despite responding well to combination therapy with Venetoclax and HMA or LDAC, persistent IDH2 mutation is commonly detectable in remission^[32]^. Although infrequent, new IDH2 mutation has been found at the time of relapse^[84]^, raising questions if adding IDH inhibitor as part of maintenance or consolidative therapy would result in deeper remissions and longer survival. Future studies are needed to address these questions. While NPM1 mutation is generally eliminated by Venetoclax-based therapies, concurrent mutation with FLT3-ITD may confer resistance^[32,65]^. As NPM1 mutation is associated with heightened HOX/MEIS1 signature^[32]^, the possible role of menin inhibitor merits further investigation and has been shown to enhance anti-AML efficacy of Venetoclax in NPM1-mutant AML models^[86]^. A combination of menin inhibitor (DS-1594) with Azacitidine and Venetoclax will be evaluated for NPM1c mutated R/R AML in an ongoing phase 1/2 clinical trial (NCT04752163)^[65]^. 

In keeping with the response seen with Venetoclax monotherapy, patients with spliceosomal mutation (SRSF2, U2AF1, SF3B1, ZRSR2) obtained a higher response rate to Venetoclax and HMA therapy compared to wild-type patients (CRi/CRh of 28% *vs.* 11%)^[87]^. Interestingly, the presence of co-mutation seems to influence differential responses seen among genes encoding spliceosome complex. SRSF2 appeared to be enriched with IDH mutations, especially IDH2, compared to the rest of spliceosomal genes cohort^[87]^. The impressive outcome was seen among SRSF2/IDH1/2 mutated patients with 1-year OS of 100% and 2-year OS of 88%^[87]^. Patients with SRSF2/IDH1/2 co-mutation also had statistically significant higher survival compared to SRSF2 mutated patients without concurrent IDH mutation, further substantiating the important presence of IDH mutation in driving Venetoclax sensitivity in the SRSF2 group^[87]^. On the other hand, U2AF1 mutation was enriched with RAS mutation with lower CRc and MRD negative CR rates^[87]^.

### Molecular determinants of resistance to Venetoclax combination therapies

In multiple clinical trials of Venetoclax and HMA or LDAC, TP53 mutation was found to be the predominant mutation in the AML patients that did not respond to Venetoclax-based therapy (7/20 cases)^[32]^. While most of the cases harbored a mutation in the DNA binding domain^[88,89]^, different forms of TP53 abnormalities were observed, including single mutation without TP53 deletion (deletion 17p) and multiple mutations with or without chromosomal abnormality in the TP53 or non-TP53 domain^[88]^. Complex cytogenetics was seen in the majority of TP53 mutated cases^[32,88,89]^, aligned with a known high prevalence of TP53 mutation in AML with complex karyotype^[90]^. These findings underscore TP53 mutation and complex cytogenetics as the most common cause of primary resistance to Venetoclax combination therapy.

Validating preclinical findings^[55,59,60]^, TP53 mutation was associated with significantly lower response rates to Venetoclax and Decitabine combination therapy compared to wild-type group (ORR of 66% *vs.* 89%, CR/CRi of 57% *vs.* 77%, and MRD negative rate of 29% *vs.* 59%)^[88]^. There was a trend toward improved survival in patients who responded or initially responded prior to relapse compared to primary refractory cases. In responder cases, TP53 mutated AML cohort had a shorter duration of response of 3.5 months *vs.* not reached in the TP53 wild-type AML cohort^[88]^. These lower responses translated into poor survival with 60-day mortality and median OS of 26% and 5.2 months in the TP53 mutated patients compared to 4% and 19.4 months in the wild-type patients. Interestingly, TP53 mutation burden [variant allele frequency (VAF)] was not proven to be a predictor of response in this mutation cohort, although gain in VAF may be seen at relapse^[88]^. In line with this finding, single-cell *DNA* sequencing on AML patient samples upon relapse to Venetoclax-based therapy showed expansion of clones containing TP53 mutation under the selective pressure of therapy with a Venetoclax-based regimen^[32]^. While TP53 mutation may be detected at diagnosis, expansion of new TP53 variants is frequently detected at the time of relapse, with or without concurrent structural loss of 17p locus, suggesting a selection of clones with biallelic TP53 perturbations^[32]^. Given lower response rates, shorter duration of response, and poor survival, TP53 mutated patients do not derive long-term benefits from Venetoclax-based therapies and should be offered clinical trials whenever feasible. Research to develop novel agents and treatment strategies is urgently needed given the poor prognosis with limited treatment options in this population.

A focused analysis of relapse patterns after Venetoclax-based combination therapy reported the most common emerging mutations in genes involved in signaling pathways (NF1, FLT3-ITD, NRAS, JAK1), in line with the above-mentioned preclinical findings; RNA splicing (U2AF1, U2AF2, SRSF2, ZRSR2), and transcription factors (IKZF1, SETBP1, RUNX1, STAT5A), followed by tumor suppressors (TP53, WT1), and epigenetic modifiers (BCOR, CREBBP)^[7]^. At relapse, concurrent expansion of clones with different types of activating kinase mutations was shown, including FLT3-ITD, FLT3-TKD, FLT3 N676, RAS, CBL^[32]^. The emergence of multiple new mutations was also observed in a study by Stahl *et al.*^[84]^, corroborating previous findings. These observations indicate that adaptive resistance may be governed by the complex interaction between various clones rather than single driver mutation. The polyclonal nature of the clonal expansion adds a tremendous challenge to salvage management of AML patients relapsing post Venetoclax-based induction, which is currently associated with extremely poor outcomes and median survival of less than three months^[7,32]^.

### Future directions and strategies to mitigate resistance

Current understanding of the mechanisms behind Venetoclax resistance identified in preclinical studies led to the development of several combination strategies that have entered clinical trials, as summarized in Table 1. Amongst these regimens, the combination of Venetoclax with FLT-3 tyrosine kinase inhibitors (TKIs) is under rigorous clinical investigation^[91,92]^. In a phase 1b trial, the combination of Gilteritinib and Venetoclax reached an ORR of 90% in FLT-3 mutated R/R AML, with similarly high responses in patients who failed prior TKIs^[91]^. Preliminary result from the “triplet” with Quizartinib, Decitabine and Venetoclax is promising, with a composite response rate (CRc) of 69% and median OS of 7.1 months in the R/R setting, while median OS was not reached in the frontline setting^[92]^. Recruitment is ongoing for Quizartinib as Venetoclax “doublet” (NCT03735875) or “triplet” (NCT03661307) therapy, and updated results are eagerly anticipated^[92,93]^.

**Table 1:** Clinical trials evaluating venetoclax-based combination regimens

**Drug Regimen**	**Treatment Category**	**Mutation (if required for eligibility)**	**Clinicaltrials.gov identifier**	**Target Number of patient enrollment^++^**	**Phase**	**Year of study initiation**
Azacitidine + Venetoclax	Frontline		NCT03466294	42	II	2018
Venetoclax + Decitabine	Both^+^		NCT03404193	400	II	2018
ASTX727 (Decitabine and Cedazuridine) + Venetoclax	Both^+^		NCT04657081	124	I/II	2021
ASTX727 (Decitabine and Cedazuridine) + Venetoclax	Both^+ ^		NCT04746235	40	II	2021
Venetoclax + Cladribine + LDAC induction followed by Cladribine+ LDAC + Azacitidine	Frontline		NCT03586609	85	II	2018
LDAC + Venetoclax^^	Frontline		NCT02287233	94	I/II	2014
LDAC + Venetoclax *vs* LDAC + placebo	Frontline		NCT03069352	211	III	2017
CPX-351 (Liposome-encapsulated Daunorubicin-Cytarabine) + Venetoclax	Both^+^	In RR subset, (+) RAS pathway activating mutation: KIT, HRAS/NRAS/KRAS, BRAF, CBL or PTPN11 or loss of function mutation of NF1	NCT03629171	52	II	2018
Ivosidenib (IDH1 inhibitor) + Venetoclax +/- Azacitidine	Both^+^	IDH1+	NCT03471260	30	I/II	2018
Enasidenib (IDH2 inhibitor) + Venetoclax	Both^+^	IDH2 (+)	NCT04092179	48	I/II	2020
Gilteritinib (FLT3 inhibitor) + Venetoclax^^	Salvage		NCT03625505	61	I	2018
Gilteritinib (FLT3 inhibitor) + Azacitidine + Venetoclax	Salvage	FLT3	NCT04140487	42	I/II	2019
Gilteritinib (FIT3 inhibitor) + ASTX727 (Decitabine and Cedazuridine) + Venetoclax	Salvage (phase I), Both (phase II)^+^	FLT3	NCT05010122	42	I/II	2021
Quizartinib (FLT3 inhibitor) + Venetoclax	Salvage	FLT3	NCT03735875	32	I/II	2019
Quizartinib (FLT3 inhibitor) + Decitabine + Venetoclax	Both^+^	FLT3	NCT03661307	52	I/II	2018
S64315 (MCL-1 inhibitor) + Venetoclax	Salvage		NCT03672695	40	I	2018
AZD5991 (MCL-1 inhibitor) + Venetoclax**	Salvage		NCT03218683	144	I/II	2017
Pevonedistat (NAE inhibitor) +/- Venetoclax + Azacitidine	Frontline		NCT03862157	40	I/II	2019
Cobimetinib (MEK inhibitor) + Venetoclax; Idasanutlin (MDM2 inhibitor) + Venetoclax^^	Salvage		NCT02670044	88	I	2016
Trametinib (MEK inhibitor) + Azacitidine + Venetoclax	Both^+^	(+) RAS pathway activating mutation in R/R subset	NCT04487106	40	II	2020
Dinaciclib (CDK inhibitor) + Venetoclax	Salvage		NCT03484520	48	I	2018
Alvocidib (CDK inhibitor) + Venetoclax^^	Salvage		NCT03441555	36	I	2018
CYC065 (CDK inhibitor) + Venetoclax	Salvage		NCT04017546	25	I	2019
APR-246 + Venetoclax +/- Azacitidine	Frontline	TP53 +	NCT04214860	51	I	2019
Magrolimab + Venetoclax + Azacitidine or MEC or CC-486 (oral Azacitidine)	Both^+^		NCT04778410	164	II	2021
Magrolimab + Azacitidine + Venetoclax	Frontline (phase I), Salvage (phase II)		NCT04435691	98	I/II	2021
ALX148 (Evorpacept) + Venetoclax + Azacitidine	Both^+^		NCT04755244	97	I/II	2021
TTI-622 (SIRPα-IgG4 Fc) + AZA +/-VEN	Frontline	TP53 +/-	NCT03530683	150	I	2018
Ph I/IIb DS-1594 (Menin inhibitor) +/- Azacitidine + Venetoclax or miniHCVD	Salvage	Presence of MLL rearrangement, NPM1 (+)	NCT03735875	32	I/II	2019

*MEC *Mitoxantrone, Etoposide, and Cytarabine. *vs.* ^^Completed. **Suspended. ^+^Both frontline and salvage therapy. ^++^Actual number of patients enrolled for completed studies.

MCL-1 undoubtfully plays an important role in mediating Venetoclax resistance through numerous upstream mediators, both genomic and epigenetic. Hence, targeting MCL-1 directly or indirectly is rational to improve sensitivity to Venetoclax^[38-40,46]^. The progress in replicating the success of direct MCL-1 inhibitor from bench to bedside has been hindered by observed troponin leak, possibly related to the regulation of mitochondrial homeostasis by MCL-1^[47,94,95]^. S64315, a direct MCL-1 inhibitor, is being evaluated in the early phase of clinical trial in combination with Venetoclax (NCT03672695)^[46,47,93]^, and additional data on safety profile and efficacy is anticipated.

Several approaches have been pursued to target MCL-1 indirectly, including inhibition of MCL-1 transcription by CKD9 inhibitors, upregulation of NOXA to increase MCL-1 neutralization by NEDD8-activating enzyme (NAE) inhibitor, and targeting RAS-RAF-MEK-ERK (MAPK) pathway to promote MCL-1 degradation by MEK inhibitor and MDM2 inhibitor (a P53 activator)^[53,96-98]^. Based on synergy seen in the preclinical studies^[53,96,98]^, multiple clinical trials evaluating the combination of Venetoclax and these novel small molecule inhibitors are being conducted, as summarized in Table 1. Several clinical trials have published their preliminary finding. In the newly diagnosed secondary AML population, the combination of Pevonidostat (NAE inhibitor), Azacitidine and Venetoclax (NCT03862157) was safe and efficacious with a CR/CRi rate of 70% and 6-months OS of 82%^[99]^. A phase 1 clinical trial (NCT02670044) of Venetoclax and Cobimetinib (MEK inhibitor) or Idasanutlin (MDM2 inhibitor) is completed. In R/R/ AML, a preliminary result showed an ORR of 18% in Venetoclax and Cobimetinib cohort^[100]^. However, Venetoclax 600 mg and Idasanutlin 200 mg arm achieved an ORR of 38%^[100]^. This finding is encouraging as the achieved response rate was higher compared to Venetoclax monotherapy or combination with LDAC or HMA in R/R population^[4,84]^. In terms of toxicity, gastrointestinal side effects were predominant with diarrhea, nausea, and vomiting, more with the Cobimetinib combination than idasanutlin^[100]^.

TP53 mutation confers Venetoclax resistance and is associated with worse outcomes^[55,84]^. MDM2 inhibitor activates wild-type p53 by disrupting p53-MDM2 interaction, which consequently prevents proteosomal p53 degradation, increases p53-regulated, and reduces p53 nuclear export^[53,101]^. However, MDM2 inhibitor depends on intact p53 function to exert apoptotic activity^[102]^. In the setting of mutant TP53, APR-246 (eprenetapopt) was developed^[103]^. Through covalent binding of its reactive electrophile form (methylene quinuclidinone) to mutant p53, APR-246 (methylated PRIMA-1) restores its function and induces p53-dependent apoptosis^[103]^. A combination of APR-246, Venetoclax and Azacitidine is being evaluated in phase 1 clinical trial (NCT04214860)^[93]^.

Another strategy is harnessing the innate immune system. Leukemic cells evade macrophage-mediated phagocytosis by overexpressing CD47 which binds to the signal regulatory protein alpha (SIRPα) receptor on the macrophage to produce a “do not eat me” signal^[3,104]^. CD47 becomes a potential therapeutic target as it is highly expressed on LSCs^[105]^. Several compounds have been developed to block CD47 and SIRPα interaction^[104]^. Magrolimab (Hu5F9-G4), an anti-CD47 antibody, was the first one to enter the clinical phase^[104]^. In a phase 1 study, Magrolimab was well tolerated and reduced LSC fraction in patients who achieved a response^[106]^. Anemia was a notable on-target effect managed by priming and maintenance dose^[106]^. Importantly, Magrolimab was not observed to augment Azacitidine’s toxicity when used in combination^[104,106]^. This suggests that adding another cytotoxic agent like Venetoclax in combination with Magrolimab is likely safe and synergistic due to the enhanced pro-phagocytic signal^[104,106,107]^. Clinical trials on the combination of Venetoclax and anti-CD47 antibody (Magrolimab, ALX148), or SIRPαFc fusion protein (TTI-622) are ongoing to validate the safety and efficacy of this novel immunotherapy regimen [Table 1].

Being protected by the bone marrow microenvironment (BMM), eliminating leukemia stem cells (LSCs) is challenging to any cytotoxic regimen, including Venetoclax^[108]^. In addition to protection, BMM also provides a survival signal and promotes stemness features in resistant AML cells^[109,110]^. Yu *et al.*^[110]^ recently discovered the role of CD44, an adhesion molecule expressed by LSCs, in governing Venetoclax resistance induced by stimulation of the CXCR4-CXCL12 pathway. Higher MCL-1 expression was also observed after CXCL12 stimulation, which was abrogated by CD44 knockout^[110]^. Loss of CD44 function also reduced the viability of Venetoclax resistant cells^[110]^. With several small molecule inhibitors in development^[111]^, a combination of CD44 or CXCR4-CXC12 pathway blockade with Venetoclax-based therapies may be worth pursuing.

## CONCLUSIONS

The advent of Venetoclax as a BCL-2 inhibitor has transformed AML management. Multiple Venetoclax-based combination therapies are developed based on current knowledge about mechanisms of responsiveness and resistance to Venetoclax. The heterogenous nature of the disease with rapid and divergent clonal evolution poses a unique challenge when it comes to designing an optimal maintenance or salvage therapy. Molecular profiling would be helpful in personalizing treatment plans, especially in selecting a frontline regimen and prior to changing therapy upon MRD persistence, refractoriness or disease relapse.

Several treatment approaches are being evaluated in clinical trials, with remaining questions to be addressed in the future, all to tackle resistance and improve the durability of response. Current strategies are directed toward maximizing induction therapy by the introduction of “triplets” therapy which incorporates several novel targeted therapies to produce a deeper response upfront with the hope to prevent the emergence of mutation from the surviving clone. Sequential therapy is also of great interest, which can be done empirically [such as Cladribine + LDAC + Venetoclax alternating with Azacitidine+Venetoclax (NCT03586609)] or adaptively tailored to the molecular signature of the resistance clones. Each approach, however, comes with its own challenge. Triplets therapy may only be suitable for selected genomic subsets and may require Venetoclax’s dose adjustment to avoid prolonged myelosuppression. Adaptive sequential therapy is an attractive option. However, this strategy requires sensitive molecular techniques for early detection of rising clone(s), which are not readily available at the moment. Reliance on the limited targetable options further hinders its applicability. Hence, the most feasible option currently is a “shot-gun” combo approach aiming at avoiding resistance through the rotating nature of chemo- or immune-therapy agents with different mechanisms of action, aided by Venetoclax as a universal sensitizer.

Considering these limitations, intensive multi-agent chemotherapy and Venetoclax combination would be the best approach for fit younger AML patients currently as a bridge to stem cell transplantation. Immune-based therapy is another promising approach, given its theoretical efficacy across genomic subsets. Current research is focusing on the benefit of immune-based therapy as “MRD erasers” in maintenance or consolidation therapy in patients with a low disease burden^[2,3]^. Several agents are currently in development, such as bispecific T-cell engagers (BiTEs), NK engagers, cancer antigen vaccines, and cellular therapies^[2,3]^. With multiple novel agents added to the AML armamentarium, additional questions remained unanswered, including the efficacy and duration of response of new combination regimens, the molecular pattern of drug response and resistance, duration of therapy after achieving response to treatment, and response to prior regimen after therapy interruption or discontinuation. Future research would be crucial in addressing these questions to evaluate how to optimally use these different types of therapeutic strategies and to identify patients that benefit the most.

## DECLARATIONS

### Copyright

© The Author(s) 2022.
